# *BARD1* is a Low/Moderate Breast Cancer Risk Gene: Evidence Based on an Association Study of the Central European p.Q564X Recurrent Mutation

**DOI:** 10.3390/cancers11060740

**Published:** 2019-05-28

**Authors:** Malwina Suszynska, Wojciech Kluzniak, Dominika Wokolorczyk, Anna Jakubowska, Tomasz Huzarski, Jacek Gronwald, Tadeusz Debniak, Marek Szwiec, Magdalena Ratajska, Katarzyna Klonowska, Steven Narod, Natalia Bogdanova, Thilo Dörk, Jan Lubinski, Cezary Cybulski, Piotr Kozlowski

**Affiliations:** 1 Department of Molecular Genetics, Institute of Bioorganic Chemistry, Polish Academy of Sciences, 61704 Poznan, Poland; kklonowska@bwh.harvard.edu; 2 Department of Genetics and Pathology, International Hereditary Cancer Center, Pomeranian Medical University in Szczecin, 71252 Szczecin, Poland; kluzniak.w@gmail.com (W.K.); dominikawok@o2.pl (D.W.); aniaj@pum.edu.pl (A.J.); huzarski@pum.edu.pl (T.H.); jgron@pum.edu.pl (J.G.); debniak@sci.pum.edu.pl (T.D.); lubinski@pum.edu.pl (J.L.); cezarycy@sci.pum.edu.pl (C.C.); 3 Independent Laboratory of Molecular Biology and Genetic Diagnostics, Pomeranian Medical University in Szczecin, 71252 Szczecin, Poland; 4 Department of Clinical Genetics and Pathology, University of Zielona Gora, 65046 Zielona Gora, Poland; 5 Department of Surgery and Oncology, University of Zielona Gora, 65046 Zielona Gora, Poland; szwiec72@gmail.com; 6 Department of Biology and Medical Genetics, Medical University of Gdansk, 80211 Gdansk, Poland; magdalena.ratajska@gumed.edu.pl; 7 Women’s College Research Institute, Women’s College Hospital, Toronto, ON M5S 1B2, Canada; steven.narod@wchospital.ca; 8 Dalla Lana School of Public Health, University of Toronto, Toronto, ON M5T 3M7, Canada; 9 Radiation Oncology Research Unit, Department of Radiation Therapy and Special Oncology, Hannover Medical School, Hannover, 30625 Lower Saxony, Germany; bogdanova.natalia@mh-hannover.de; 10 Gynaecology Research Unit, Department of Obstetrics and Gynecology, Hannover Medical School, Hannover, 30625 Lower Saxony, Germany; Doerk.Thilo@mh-hannover.de

**Keywords:** breast cancer, *BARD1*, genotyping, p.Q564X, p.R658C, p.R659R, breast cancer risk

## Abstract

In addition to several well-established breast cancer (BC) susceptibility genes, the contribution of other candidate genes to BC risk remains mostly undefined. *BARD1* is a potentially predisposing BC gene, however, the rarity of its mutations and an insufficient family/study size have hampered corroboration and estimation of the associated cancer risks. To clarify the role of *BARD1* mutations in BC predisposition, a comprehensive case-control association study of a recurring nonsense mutation c.1690C>T (p.Q564X) was performed, comprising ~14,000 unselected BC patients and ~5900 controls from Polish and Belarusian populations. For comparisons, two *BARD1* variants of unknown significance were also genotyped. We detected the highest number of *BARD1* variants in BC cases in any individual *BARD1*-specific study, including 38 p.Q564X mutations. The p.Q564X was associated with a moderately increased risk of BC (OR = 2.30, *p* = 0.04). The estimated risk was even higher for triple-negative BC and bilateral BC. As expected, the two tested variants of unknown significance did not show significant associations with BC risk. Our study provides substantial evidence for the association of a deleterious *BARD1* mutation with BC as a low/moderate risk allele. The p.Q564X was shown to be a Central European recurrent mutation with potential relevance for future genetic testing.

## 1. Introduction

Breast cancer (BC) is the most common cancer diagnosed in women worldwide, with over 1.5 million new cases diagnosed each year [1]. Most BCs occur sporadically, potentially as a result of interactions between multiple environmental, lifestyle, hormonal and genetic factors. However, approximately 5–10% of BCs present with a familial aggregation due to hereditary mutations in highly penetrant genes. Familial BC frequently co-occurs and shares some genetic background with epithelial ovarian cancer (OC). Other indicators of hereditary BC are younger age of onset, bilateral disease, and triple-negative BC (TNBC). Mutations in high-risk genes, including *BRCA1* [2] and *BRCA2* [3], as well as in a moderate-risk *PALB2* gene [4], are responsible for approximately half of familial BC cases. Mutations in *CDH1* [5], *PTEN* [6], and *TP53* [7], which are associated with hereditary diffuse cancer, Cowden disease, and Li-Fraumeni syndrome, respectively, also contribute to high-risk predisposition of BC but are very rare. Low-penetrance genes, common polymorphisms [8,9], copy number variants [10,11,12], and epigenetic alterations [13,14] on familial BC risk are also suggested as constitutive risk factors but do not explain the missing fraction of familial heritability.

Genes encoding proteins that interact with BRCA1 and BRCA2 in different DNA damage response and tumor suppressor processes are among the candidate BC and/or OC susceptibility genes. One such gene that has been intensively studied is *BRCA1-associated RING domain 1* (*BARD1*) [15], because the BARD1 protein shares both structural and functional similarities with BRCA1 and because the interaction between BARD1 and BRCA1 plays an important role in maintaining the stability and manifestation of the tumor suppressor function of BRCA1 [16].

In humans, the *BARD1* gene spans a region of ~80 kb on the long arm of chromosome 2 (2q34-35) and comprises 11 exons. The gene encodes a protein of 777 amino acids that contains one N-terminal RING-finger domain, two C-terminal tandem BRCT domains (homologous to corresponding domains in BRCA1), and three Ankyrin (ANK) repeat domains. BARD1 may act as a tumor suppressor in the BRCA1-dependent pathways, which is associated with specific BRCA1/BARD1 heterodimer formation via N-terminal RING-finger domains. The BRCA1/BARD1 heterodimer demonstrates ubiquitin ligase activity, which functions in DNA damage response pathways, cell cycle regulation, and chromatin structural and hormone signaling modulation (for review see [17,18]). Because BARD1 may interact with other molecules implicated in genome integrity, a BRCA1-independent tumor suppressor functions for BARD1 have also been suggested. Examples include the interaction between BARD1 and p53, which stabilizes p53, facilitates p53 phosphorylation, and induces p53 apoptotic activity in response to DNA damage [19,20].

The hitherto reported *BARD1* mutation screening studies of familial and unselected BC and/or OC cases has led to the identification of numerous *BARD1* sequence variants [21,22,23,24,25,26,27,28,29,30,31,32,33,34,35,36,37,38,39,40,41,42,43,44,45,46,47,48,49,50,51,52,53,54]. Additionally, *BARD1* common variants have been associated with an aggressive subset of human neuroblastomas [55,56], lung cancer [57] and colon cancer [58]. *BARD1* mutations identified in BC cases include deleterious and potentially deleterious mutations that lead to premature termination of translation, disruption of protein structure/function, or alternative splicing. Some of these mutations have been shown to cosegregate in families with cancer [22,24,33]. Despite these observations, none of the abovementioned studies (including our own [36,37]) have provided strong, statistically supported data for the role of *BARD1* mutations in cancer predisposition. This lack of data is due mostly to the following limitations: (i) most of the studies were not performed in a case-control manner (i.e., the control samples were either not screened at all or not analyzed in the same way as the case samples), (ii) a relatively small group of patients were analyzed (usually 100–300), and consequently, a small number of mutations were identified in individual studies, preventing reliable risk estimates, and (iii) in cases of mutations identified in families, the families were too small to perform formal linkage (mutation-cancer co-segregation) analysis. More recently, several studies reporting results for targeted sequencing with cancer-predisposition gene panels, including *BARD1*, have been published [35,39,40,41,42,43,44,45,46,47,48,49,50,51,52,59,60,61,62,63,64,65,66,67,68,69,70,71,72,73]. The large-scale and cumulative character of some of these studies, including our own meta-analysis [74], allowed us to estimate the *BARD1*-attributed BC risk. However, in most of these studies, the risk was estimated by comparisons of mutation frequency in BC cases to that in controls from publicly available databases, not well matched in terms of the population studied (geographical region) and/or the methodology used. Such analysis may be affected by population stratification, e.g., due to an unequal distribution of founder mutations, which frequently occur in BC predisposition genes. Due to insufficient evidence of a *BARD1* association with BC risk, *BARD1* was not included in the recently proposed consensus multigene panel by the UK Cancer Genetics Group for BC testing [75]. Also, although *BARD1* was included in the most recent version of “The National Comprehensive Cancer Network (NCCN) Guidelines for Genetic/Familial High-Risk Assessment: Breast and Ovarian” as a gene that may be linked with increased breast cancer risk, still due to insufficient evidence, no management recommendations for women with a pathogenic *BARD1* variant was provided at this time.

To overcome the abovementioned limitations, we took advantage of a deleterious nonsense mutation, namely, c.1690C>T (p.Q564X), identified in the Polish population in several independent studies [36,37,76], to provide a reliable estimation of BC risk associated with *BARD1* mutations. We performed an association analysis of the mutation in two large cohorts recruited from the POLISH and closely related BELARUSIAN populations, by genotyping ~14,000 BC patients and ~5900 control samples. Additionally, in the POLISH group, we also analyzed two rare *BARD1* variants of unknown significance (also recurring in the Polish population), a missense variant c.1972C>T (p.R658C) and a synonymous variant c.1977A>G (p.R659R). The analysis showed that the definitively deleterious *BARD1* mutation is associated with low/moderate BC risk (OR ~2) and that the risk is further increased in the group of patients with a risk for heritable BC (OR ~3), including TNBC, bilateral BC, early diagnosis of BC, and familial BC/OC.

## 2. Results

### 2.1. Selection of BARD1 Variants

For our genetic association study, we selected three *BARD1* sequence variants: (i) a deleterious nonsense mutation p.Q564X, located in exon VIII, and two variants of unknown significance, (ii) missense variant p.R658C, located in exon X, and (iii) synonymous variant p.R659R, located in exon X. Based on preliminary results, we estimated the frequency of occurrence of each variant in the Polish population to be ~0.3%, ~0.5%, and ~0.4%, respectively. The ClinVar status of the p.Q564X mutation is defined as pathogenic, while both the p.R658C and p.R659R variants are listed as benign/likely benign/uncertain significance [77]. At present, according to different databases, the frequency of these variants in the European population ranges from 0.01–0.02%, 1.56–1.78%, and 0.59–1.02%, respectively (Table 1). As shown in Table 1, the variants of unknown significance also occur in non-European populations, while the frequency of the nonsense mutation is limited to European populations.

### 2.2. The Frequency of BARD1 Variants and BC Risk Estimates

All selected variants were screened in 12,476 BC cases and 4707 controls from the Polish population (POLISH group). Additionally, the p.Q564X mutation was screened in 1459 BC cases and 1189 controls from the Belarusian population (BELARUSIAN group). The size of the study predicted almost 100% and ~80% power to detect the effect of the mutation at the level of an OR of 3 and 2, respectively. The p.Q564X mutation was identified in 34 cases (0.27%) and seven controls (0.15%) in the POLISH group (OR = 1.83, 95%CI: 0.81–4.14, *p* = 0.14) and in four cases (0.27%) and 0 controls (0%) in the BELARUSIAN group.

A cumulative OR equals 2.30 (95%CI: 1.03–5.15, *p* = 0.04), which classifies *BARD1* as a low/moderate BC susceptibility gene (Table 2). The stratification of BC patients into clinically defined BC subtypes showed an even higher risk for TNBC (16.1% of BC patients; OR = 3.62, 95%CI: 1.21–10.78, *p* = 0.02) and bilateral BC (4.4% of BC patients; OR = 5.10, 95%CI: 1.31–19.78, *p* = 0.02). The cumulative OR for the group of patients with increased risk of hereditary BC, including TNBC, bilateral BC, early diagnosis, and/or familial BC/OC, was 2.94 (95%CI: 1.21–7.14, *p* = 0.02) (Table 3). The indicators of hereditary BC, namely, younger age at diagnosis (≤40 years) and positive family history for BC and/or OC, were also associated with increased BC risk, though individually, these associations were not statistically significant due to the low number of cases. As shown in Table 2; Table 3, adjustment for the studied cohorts did not substantially change the statistical estimates. The frequencies of the missense variant p.R658C and the synonymous p.R659R variant did not differ significantly between the analyzed the BC case and control groups (80/12476, 0.64% vs. 26/4707, 0.55%, and 49/12476, 0.39% vs. 14/4707, 0.30%, respectively), suggesting a lack of their association with BC (OR = 1.16 and OR = 1.32, respectively).

**Figure 1:**
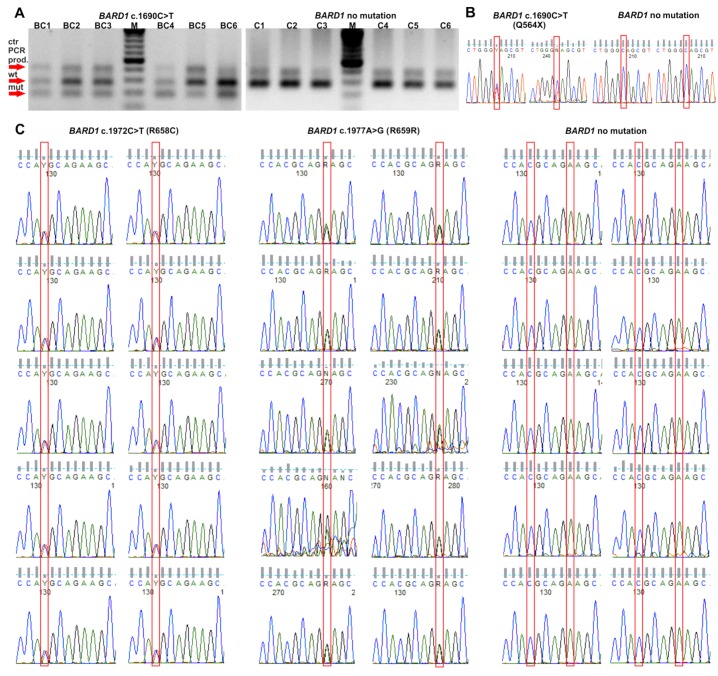
Validation of *BARD1* variants. (**A**) Validation of p.Q564X *BARD1* mutation in randomly selected carriers and noncarriers with the tetra-primer ARMS-PCR assay [BC: breast cancer patients; C: controls; M: marker, GeneRuler 100 bp Plus DNA Ladder (Thermo Fisher Scientific, Waltham, MA, USA)] and (**B**) with Sanger sequencing. (**C**) Validation of p.R658C and p.R659R *BARD1* variants in randomly selected carriers and noncarriers with Sanger sequencing. See the Materials and Methods section for details.

None of the analyzed subjects was a carrier of more than one of the tested variants. Validation of the genotyping results performed with alternative techniques in the limited number of positive and negative subjects confirmed all detected variants and proved 100% sensitivity and specificity of the genotyping results (see Section 4 and Figure 1).

### 2.3. Predictive Value of BARD1 Variants

In the next step, we compared the frequencies of selected variants in the different BC subgroups. Carriers and noncarriers of the p.Q564X mutation have a similar average age at diagnosis (54.8 and 54.1 years, respectively). As shown in Table S1, there was only a small nonsignificant increase in the mutation frequency in patients with vs. without a family history of BC and/or OC (0.23% vs. 0.30%, respectively). In the assessment of the molecular BC subtype, a higher incidence of the mutation was found in the patients with a progesterone receptor-negative (PR−) BC (0.55% vs. 0.24%, *p* = 0.03), estrogen receptor-negative (ER−) BC (0.43% vs. 0.29%) and TNBC (0.54% vs. 0.33%) than in the corresponding patients with a receptor-positive BC. However, due to the low number of cases in the subgroups, only the difference between the PR− and PR+ BC patients was significant. There was also no difference in p.Q564X mutation prevalence between the human epidermal growth factor receptor 2-positive (HER2+) and HER2-negative (HER−) BC patients.

There were no evident changes in the p.R658C and p.R659R prevalences after stratifying BC patients in terms of different clinical features (Table S2). The exceptions were a higher frequency of the p.R659R variant in patients with PR+ than in patients with PR- BC (0.44% vs. 0.17%, 0.07) and a higher frequency of p.R659R in patients with tubulolobular BC than in patients with other histological types of BC (1.72% vs. 0.30%, *p* = 0.05), however, these results were only borderline significant and may be accidental due to the small number of patients in each group. We also noticed a higher incidence of p.R658C variants among deceased patients than in those who were still alive (1.02% vs. 0.57%, *p* = 0.03).

Besides the presented data on population frequency and case-control study, we analyzed functional significance of the selected variants with the use of a wide panel of bioinformatics tools evaluating a potential effect of the particular variants on evolutionary conservation of an altered amino acid, RNA structure, or structure/function of a protein (Table S3).

## 3. Discussion

Since the discovery of *BARD1* about 20 years ago [15], many studies have been carried out to try to understand its role in cancer. Because BARD1 functions in the same molecular pathways as BRCA1, *BARD1* is considered a good candidate as a BC susceptibility gene.

The identification of recurrent *BARD1* variants in the Polish population [36,37,76] motivated us to thoroughly examine the association between *BARD1* and BC, as a founder-mutation-based study is likely to be successful in the search for and confirmation of infrequently mutated BC susceptibility genes. Similar approaches have been applied previously to investigate the contributions of recurrent variants to conferring cancer risk. The examples include the genotyping of c.5932G>T (p.E1978X) in *ATM* [78], c.1667_1667+3delAGTA in *RECQL* [79], c.509_510delGA [80,81], and c.172_175delTTGT in *PALB2* [81], for which the studies revealed associations with an elevated BC risk, and c.576+1G>A in *RAD51D* [82], which was associated with OC risk. To our knowledge, this is the first study of BC risk attributed to individual *BARD1* variants. It is important as the type and location of a particular mutation may alter the cancer risk estimate, as was previously shown for *BRCA1*/*2* mutations [83,84,85,86,87].

Considering the interaction between BARD1 and BRCA1 and the similarities in their structures, it is surprising that *BARD1* mutations are relatively rare in BC patients compared to *BRCA1* mutations. In spite of this, some of the studies for identified *BARD1* mutations suggested a weak association with BC, although individual studies have been largely unsuccessful to provide convincing and statistically supported proof of this association. This may due to an insufficient sample/study size and a lack of a geographically matched population controls, complicating the interpretation of the results. For rare mutations of lower penetrance, which have a “low/moderate” effect on phenotype, it is especially difficult to demonstrate their causative nature and thereby confirm their contributions to BC, therefore, a very large case-control analysis should be performed. As whole exome or even whole gene analysis can be expensive for a sufficient number of cases and controls, the analysis of founder mutations may be the more cost-efficient solution.

In this large case-control study, we provided strong evidence that *BARD1* is a low/moderate cancer susceptibility gene. The large collection of BC cases and controls allowed us to uncover an approximately two-fold increase in BC risk (OR = 2.3, *p* = 0.04) associated with the presence of the p.Q564X mutation. The analysis also showed that p.Q564X is recurrent, potentially a founder mutation, at least in Polish and Belarusian populations. It has to be noted, however, that further haplotype analyses are needed to prove the founder effect of the Q564X mutation. The p.Q564X frequency determined here was 25× and 14× higher in the Polish BC patients and controls, respectively, than in the gnomAD Non-Finnish European population [88] (0.27% and 0.15% vs. 0.011%, respectively) (see Table 1 and Table 2). The mutation seems to be less frequent (was not detectable) in other control populations. Additionally, the recent conference report of the German Consortium for Hereditary Breast and Ovarian Cancer revealed the p.Q564X mutation in 6 out of 14 *BARD1* mutation carriers in Germany [89]. This suggests that p.Q564X may have been spread in the wider Central European population.

The cumulative loss-of-function mutation frequency of *BARD1* was reported to range between 0.12% and 0.49% in other studies analyzing at least 1000 BC patients with multigene panels (screening the whole coding sequence of *BARD1*) [42,43,45,51,59,61,74]. These results emphasize the frequent occurrence of a single p.Q564X mutation (0.27%) in the tested populations. Although other estimates of BC risk associated with *BARD1* mutations were either statistically underpowered or used imperfectly matched controls, they are largely in line with the risk estimates of this study. Thompson et al. suggested that *BARD1* mutations were associated with moderate BC risk (OR = 3.0, *p* = 0.62) by comparing 2000 *BRCA1*/*2*-negative BC patients with geographically matched controls, though not reaching statistical significance [45]. Similar results were obtained in cumulative large-scale analyses performed by Couch et al. (OR = 2.2, *p* = 0.002) [59] and Slavin et al. (OR = 3.2, *p* = 0.012) [43], as well as in our own recently published meta-analysis (OR = 2.3, *p* < 0.0001) [74].

Some evidence for a higher effect size of p.Q564X in BC (OR = 2.9, *p* = 0.02) was obtained for BC patients characterized by features associated with hereditary BC, including TNBC, bilateral BC, early diagnosis (under 40 years old) and a positive BC/OC family history. More specifically, mutation carriers have a higher risk (OR = 3.6, *p* = 0.02) of TNBC, a tumor phenotype associated with a hereditary disease cause, a more aggressive disease course, an increased recurrence risk and a poor five-year survival rate than patients with other BC subtypes. Our results are in line with the previous study by Buys et al. [61], which demonstrated that *BARD1* mutation prevalence was higher among women with TNBC (3.3%) than among women with other BC subtypes (1.7%). Also, Shimelis et al. [90] reported that pathogenic variants in *BARD1* were enriched by more than threefold in TNBC patients (0.67%) compared to non-TNBC patients (0.18%), suggesting that *BARD1* is a predominantly TNBC predisposition gene. An even higher effect sizes for the association of *BARD1* mutations with TNBC were reported in Shimelis et al. [90] (OR = 5.92) and Castera et al. [91] (OR = 11.27), although the estimates by the latter study were based on a low number of mutation carriers.

Our study also indicated a likely contribution of *BARD1* mutations to bilateral BC (OR = 4.85, *p* = 0.02), which is another indicator of hereditary BC, in addition to TNBC. This tendency was also noticed in a study by Castera et al. [91] (OR = 6.92), however, the result did not reach statistical significance. The results of our study add to previous evidence that the contributions of particular genes to the development of specific subtypes of BC may differ. The differences may not be noticeable in an overall analysis, therefore, stratification BC tumor type (e.g., bilateral BC, TNBC) should be performed to reliably estimate BC risk.

Although the prevalence of the synonymous and missense variants analyzed in our study was slightly increased in the BC group, there was no significant association, which supports their benign/likely benign/uncertain significance status in the ClinVar database. We tested these variants in addition to p.Q564X as they had been suggested to have some functional or pathological effects. For example, p.R659R was shown to impair several exonic splicing enhancer motifs in exon 10, resulting in a transcript lacking exons 2–9 (r.[=,159_1903del], p.Cys53_Trp635delinsfsX12) [36]. However, aberrant splicing occurs only in a fraction of transcripts. Also, the p.R658C variant, which affects conserved amino acids, was detected before in BC patients [33,37,76], and a partial co-segregation of the variant with BC/OC in two small families was shown [33]. Additionally, the p.R658C variant, has been shown to correlate with a risk of lung cancer (OR = 1.55) [57]. From our results, we cannot definitively assess the role of p.R659R and p.R658C in BC predisposition, however more than three-fold increases in risk would be safely excluded from the upper confidence limits in our study.

Because the *BARD1* contribution to BC risk has been determined by us to be at the borderline of low and moderate levels, it may suggest that *BARD1* mutations contribute to a polygenic model [92,93], like for mutations of lower penetrance in other genes, including *CHEK2* or *ATM*.

The number of identified *BARD1* mutation carriers is, to our knowledge, the highest reported to date in an individual *BARD1* mutation-specific study. Even though, it has to be noted that the risk estimates are only borderline significant, and more research data from large sequencing studies will be useful to validate these findings. However, as discussed above, the results obtained from our case-control study in founder populations should be very helpful to corroborate the role of *BARD1* as a breast cancer susceptibility gene.

## 4. Materials and Methods

### 4.1. Study Population

The genetic association study for the selected *BARD1* variants was performed in two Slavic populations, the POLISH group and the BELARUSIAN group. The POLISH group comprised 12,476 BC cases (unselected for familial history of the disease) and 4707 cancer-free adult female controls from the International Hereditary Cancer Center (IHCC) cohort (Szczecin, Poland). Patients were diagnosed with invasive BC between 1996 and 2012 at one of 18 different hospitals in Poland. Patients diagnosed with ductal or lobular carcinoma in situ were excluded, with the exception of patients diagnosed with ductal carcinoma in situ with microinvasion. The average age of BC diagnosis was 54 years. Information regarding family history was collected at the time of enrollment for 92.7% of IHCC patients, and 18.9% of them had at least one first- or second-degree relative with BC and/or OC. Additionally, data on the tumor histological and molecular type, disease bilaterality, and lymph node status were collected, when possible. 

The BELARUSIAN group comprised 1459 BC patients unselected for familial history of the disease and 1189 cancer-free female controls from the Hannover-Minsk Breast Cancer Study (HMBCS). BC patients from the HMBCS were diagnosed during the years of 1998–2008 at the Belarussian Institute for Oncology and Medical Radiology Aleksandrov N.N. in Minsk or at one of the regional oncology centers in Gomel, Mogilev, Grodno, Brest, or Vitebsk, Belarus [87]. The average age of BC diagnosis was 48 years, and 29.4% of BC patients had a family history of BC and/or OC (first- or second-degree relatives). All subjects were Caucasians of European ancestry and ethnic Poles or Belarusians in the POLISH or BELARUSIAN group, respectively. Written informed consent was obtained from all subjects, and the study was approved by the appropriate local ethics committees, namely, the Institutional Review Board of Pomeranian Medical University, Szczecin, Poland; the Ethics Commission of the State Organization ‘Institute for Hereditary Diseases’, Ministry of Health, Republic of Belarus; and the Institutional Review Board at Hannover Medical School, Hannover, Germany. The ethical approval for: (i) the POLISH group has the identifier no. BN-001/33/04 (23 February 2004; Pomeranian Medical University, Szczecin, Poland) and for (ii) the BELARUSIAN group has the identifier no. 6079 (last renewal 9 February 2016; Hannover Medical School, Hannover, Germany). More detailed characteristics of the BC patients and tumors in both the POLISH and BELARUSIAN groups are provided in Table 4.

### 4.2. Variant Screening

Genotyping of the *BARD1* variants in the POLISH group was performed with the TaqMan assay, according to general recommendations, utilizing the LightCycler^®^ Real-Time PCR 480 System (Roche Life Science, Penzberg, Germany). Genotyping of the p.Q564X mutation in the BELARUSIAN group was accomplished using allele-specific SNP-type assays and Dynamic Arrays on a Fluidigm Biomark platform (Fluidigm, South San Francisco, CA, USA). The TaqMan primer and probe sets are shown in Table S4.

### 4.3. Validation of Genotyping Results

A small group of samples, which were either positive or negative for the presence of a particular variant, from both the BC and control series, were reanalyzed with alternative methods. The p.Q564X mutation identified in POLISH samples was validated with the tetra-primer amplification refractory mutation system PCR (tetra-primer ARMS-PCR) assay, designed and performed according to previously published guidelines [94]. The analysis was performed in 18 randomly selected carriers (13 from the BC group and 5 from the control group) and 20 noncarriers from the control group. The results were independently validated by Sanger sequencing analysis on an ABI Prism 3130 genetic analyzer (Applied Biosystems, Carlsbad, CA, USA) according to the manufacturer’s general recommendations. The BELARUSIAN p.Q564X mutation carriers were validated by Sanger sequencing. The p.R658C and p.R659R variants were reanalyzed in 10 carriers (for each mutation) and 10 noncarriers by Sanger sequencing. The primer sets used for confirmation of selected *BARD1* variants are shown in Table S5.

### 4.4. Statistical Analysis

The association between *BARD1* variants and BC risk was assessed using odds ratios (ORs) and 95% confidence intervals (CIs) derived from univariate logistic regression models. When combining the POLISH and BELARUSIAN groups, appropriate OR adjustments were applied. The significance of the differences between the variant carrier and noncarrier groups was determined using Fisher’s exact test and the X2 test, as appropriate for category size. All statistical tests were two-sided, and a *p* = 0.05 was considered to be the significance threshold. Analyses were performed using MedCalc Statistical Software version 18.11 (MedCalc Software bvba, Ostend, Belgium; http://www.medcalc.org).

## 5. Conclusions

This is the first study of individual *BARD1* variants in the context of BC risk. It shows that deleterious *BARD1* mutations contribute to low/moderate BC risk, thus confirming the role of *BARD1* in BC predisposition. An even higher effect size of the *BARD1* mutations was observed for TNBC and bilateral BC, i.e., subgroups attributed to hereditary BC. Our results also indicate that the BC-predisposing role of variants of unknown significance should be interpreted with caution and that effect of these mutations need to be proven individually before consideration in BC risk evaluations.

## Figures and Tables

**Table 1:** The frequencies of selected *BARD1* variants in European and other populations.

Population	Database (Population)	p.Q564X M+/N (%)	p.R658C M+/N (%)	p.R659R M+/N (%)
European	EVS (European American)	0/4300 (−)	67/4300 (1.558)	28/4300 (0.651)
	FLOSSIES (European)	1/7325 (0.014)	130/7325 (1.775)	75/7325 (1.024)
	ExAC (European non-Finnish)	6/33,368 (0.018)	535/33,352 (1.604)	196/33,357 (0.588)
	gnomAD (European non-Finnish)	7/64,526 (0.011)	1078/64,546 (1.670)	397/64,564 (0.615)
Other	gnomAD (All populations)	7/141,343 (0.005)	2302/141,376 (1.628)	575/141,400 (0.407)
	gnomAD (European Finnish)	0/12,543 (−)	316/12,557 (2.516)	33/12,559 (0.263)
	gnomAD (Ashkenazi Jewish)	0/5184 (−)	0/5183 (−)	81/5183 (1.563)
	gnomAD (East Asian)	0/9977 (−)	224/9977 (2.245)	0/9977 (−)
	gnomAD (South Asian)	0/15,307 (−)	124/15,306 (0.810)	1/15,306 (0.007)
	gnomAD (African)	0/12,481 (−)	59/12,481 (0.473)	9/12,484 (0.072)
	gnomAD (Latino)	0/17,719 (−)	444/17,718 (2.506)	30/17,719 (0.169)
	gnomAD (Other)	0/3606 (−)	57/3608 (1.580)	24/3608 (0.665)

EVS, Exome Variant Server; ExAC, Exome Aggregation Consortium; gnomAD, The Genome Aggregation Database; M+, number of subjects with a particular variant; N, number of all analyzed subjects.

**Table 2:** The frequencies and effect sizes of the three tested *BARD1* variants.

Study Population	Group	p.Q564X				p.R658C				p.R659R			
		M + /N (%)	OR	95%CI	*p*-Value	M+/N (%)	OR	95%CI	*p*-Value	M+/N (%)	OR	95%CI	*p*-Value
Controls	P	7/4707 (0.15)	-	-	-	26/4707 (0.55)	-	-	-	14/4707 (0.30)	-	-	-
	B	0/1189 (0.00)	-	-	-	-	-	-	-	-	-	-	-
	P + B	7/5896 (0.12)	-	-	-	-	-	-	-	-	-	-	-
All BC patients	P	34/12,476 (0.27)	1.83	0.81–4.14	0.14	80/12,476 (0.64)	1.16	0.75–1.81	0.51	49/12,476 (0.39)	1.32	0.73–2.40	0.36
	B	4/1459 (0.27)	n.a.	n.a.	n.a.	-	-	-	-	-	-	-	-
	P + B	38/13,935 (0.27)	**2.30**	**1.03–5.15**	**0.04**	-	-	-	-	-	-	-	-
			2.24 *	0.99–5.03 *	0.05 *		-	-	-		-	-	-
			2.12 **	0.97–4.62 **	0.06 **		-	-	-		-	-	-

**Table 3:** Prevalence of *BARD1* variants and the associated BC risk in the groups of patients stratified by BC subtypes.

Feature	Group	p.Q564X				p.R658C				p.R659R			
		M+/N (%)	OR	95%CI	*p*-Value	M+/N (%)	OR	95%CI	*p*-Value	M+/N (%)	OR	95%CI	*p*-Value
TNBC	P	6/1120 (0.54)	3.62	1.21–10.78	0.02	6/1120 (0.54)	0.97	0.40–2.36	0.95	2/1120 (0.18)	0.60	0.14–2.64	0.50
Bilateral BC	P	2/447 (0.45)	3.02	0.63–14.57	0.17	4/447 (0.89)	1.63	0.56–4.68	0.37	0/447 (0.00)	0.36	0.02–6.07	0.48
	P + B	3/498 (0.60)	**5.10**	**1.31–19.78**	0.02	-	-	-	-	-	-	-	-
			4.85 *	1.24–18.93 *	0.02 *		-	-	-		-	-	-
			5.22 **	1.50–18.19 **	0.01 **		-	-	-		-	-	-
BC diagnosed ≤40 y.o.	P	4/1286 (0.31)	2.09	0.61–7.17	0.24	7/1286 (0.54)	0.99	0.43–2.28	0.97	5/1286 (0.39)	1.30	0.47–3.64	0.61
	P + B	5/1640 (0.30)	**2.57**	**0.82–8.12**	**0.11**	-	-	-	-	-	-	-	-
			2.60 *	0.82–8.21 *	0.10 *		-	-	-		-	-	-
			2.66 **	0.88–8.03 **	0.08 **		-	-	-		-	-	-
≥1 BC/OC relatives	P	5/2191 (0.23)	1.54	0.49–4.84	0.46	14/2191 (0.64)	1.16	0.60–2.22	0.66	3/2191 (0.14)	0.46	0.13–1.60	0.22
	P + B	6/2620 (0.23)	**1.93**	**0.65–5.75**	**0.24**	-	-	-	-	-	-	-	-
			1.88 *	0.63–5.60 *	0.26 *		-	-	-		-	-	-
			1.92 **	0.67–5.46 **	0.22 **		-	-	-		-	-	-
“Hereditary” BC risk patients ***	P	13/4130 (0.31)	2.12	0.85–5.32	0.11	30/4130 (0.73)	1.32	0.78–2.23	0.31	9/4130 (0.22)	0.73	0.32–1.69	0.47
	P + B	16/4600 (0.35)	**2.94**	**1.21–7.14**	**0.02**	-	-	-	-	-	-	-	-
			2.93 *	1.20–7.19 *	0.02 *		-	-	-		-	-	-
			2.77 **	1.18–6.49 **	0.02 **		-	-	-		-	-	-

Table 2 & Table 3: P: POLISH group; B: BELARUSIAN group; P + B: POLISH and BELARUSIAN GROUP; M+: number of patients with a particular variants; N: number of all genotyped patients; y.o.: years old; *: adjusted for the origin of the study; **: a Mantel-Haenszel method under the fixed effects model (please note that this analysis may be biased due to no mutations in the BELARUSIAN control group); ***: TNBC^#^ and/or bilateral BC and/or BC diagnosed ≤40 y.o. and/or ≥1 BC/OC relative; #: TNBC status was not determined in the BELARUSIAN group.

**Table 4:** Clinical characteristics of the BC patients.

Feature	POLISH Group *n* = 12,476		BELARUSIAN Group *n* = 1459	
	*n* of Cases in Which Feature Status was Determined	*n* and (%) of Positive Cases	*n* of Cases in Which Feature Status was Determined	*n* and (%) of Positive Cases
Age at diagnosis (years)				
≤40	12,459	1286 (10.3)	1459	354 (24.3)
41–50	12,459	4394 (35.3)	1459	520 (35.6)
51–60	12,459	3248 (26.1)	1459	329 (22.5)
61–70	12,459	2226 (17.9)	1459	176 (12.1)
≥71	12,459	1305 (10.5)	1459	80 (5.5)
Number of relatives with BC				
0	11,564	9593 (83.0)	1459	1214 (83.2)
1	11,564	1508 (13.0)	1459	236 (16.2)
2	11,564	344 (3.0)	1459	9 (0.6)
≥3	11,564	119 (1.0)	1459	0 (0.0)
Numbers of relatives with OC				
0	11,564	11,263 (97.4)	1459	1457 (99.9)
≥1	11,564	301 (2.6)	1459	2 (0.1)
Histological type of BC				
Ductal, grade 3	8670	1998 (23.0)	525	42 (8.0)
Ductal, grade 1–2	8670	4057 (46.8)	525	179 (34.1)
Ductal, grade unknown	8670	664 (7.7)	525	165 (31.4)
Medullary	8670	287 (3.3)	525	10 (1.9)
Lobular	8670	1243 (14.3)	525	108 (20.6)
Tubulolobular	8670	116 (1.3)	525	21 (4.0)
DCIS with microinvasion	8670	305 (3.5)	525	0 (0.0)
Molecular type of BC				
Oestrogen receptor-positive	8572	5970 (69.6)	875	676 (77.3)
Progesterone receptor-positive	8270	5885 (71.2)	n.d.	n.d.
HER2-positive	7215	1270 (17.6)	n.d.	n.d.
TNBC	6937	1120 (16.1)	n.d.	n.d.
Size (cm)				
<1	7909	914 (11.6)	n.d.	n.d.
1–1.9	7909	3204 (40.5)	n.d.	n.d.
2–4.9	7909	3462 (43.8)	n.d.	n.d.
≥5	7909	329 (4.2)	n.d.	n.d.
Other features				
Bilateral BC	9787	447 (4.6)	1459	51 (3.5)
Lymph node-positive	8162	3551 (43.5)	n.d.	n.d.
Vital status (deceased)	12,349	2066 (16.7)	n.d.	n.d.

HER2, human epidermal growth factor receptor 2; n.d., not determined.

## Supplementary Materials

The following are available online at https://www.mdpi.com/2072-6694/11/6/740/s1, Table S1: Characteristics of BC patients in terms of different clinical features and the status of the p.Q564X *BARD1* mutation (carrier vs. noncarrier), Table S2: Characteristics of BC patients in terms of different clinical features and the status of p.R658C and p.R659R *BARD1* variants (carriers vs. noncarriers), Table S3: The computational analyses of the *BARD1* variants selected for the analysis, Table S4: Primers and probes used for *BARD1* variants genotyping with the TaqMan assay, Table S5: Primers used for validation of *BARD1* variants.
