# Supplementary material for: BARD1 is a Low/Moderate Breast Cancer Risk Gene: Evidence Based on an Association Study of the Central European p.Q564X Recurrent Mutation
Source: Cancers (Basel). 2019 May 28;11(6):740. doi: 10.3390/cancers11060740 (PMC6627038; doi:10.3390/cancers11060740)
Supplement: Supplementary file 1 [file cancers-11-00740-s001.pdf]

SUPPLEMENTARY TABLES

Table S1. Characteristics of BC patients in terms of different clinical features and the status of the p.Q564X *BARD1* mutation (carrier vs noncarrier).

feature	Group	YES	NO	p-value
		M+/M- (%)	M+/M- (%)	Fisher/χ2
Histological type of BC				
Ductal, grade 3	P	10/1988 (0.50)	18/6654 (0.27)	0.118/0.111
	B	0/42 (0.00)	3/480 (0.62)	1.000/0.609
	P+B	10/2030 (0.49)	21/7134 (0.29)	0.193/0.176
Ductal, grade 1-2	P	12/4045 (0.30)	16/4597 (0.35)	0.709/0.676
	B	1/178 (0.56)	2/344 (0.58)	1.000/0.978
	P+B	13/4223 (0.31)	18/4941 (0.36)	0.720/0.644
Ductal, grade unknown	P	2/662 (0.30)	26/7980 (0.32)	1.000/0.918
	B	0/165 (0.00)	3/357 (0.83)	0.555/0.240
	P+B	2/827 (0.24)	29/8337 (0.35)	1.000/0.618
Medullary	P	2/285 (0.70)	26/8357 (0.31)	0.237/0.256
	B	0/10 (0.00)	3/512 (0.58)	1.000/0.809
	P+B	2/295 (0.67)	29/8869 (0.33)	0.265/0.310
Lobular	P	1/1242 (0.08)	27/7400 (0.36)	0.170/0.104
	B	2/106 (1.85)	1/416 (0.24)	0.109/0.048
	P+B	3/1348 (0.22)	28/7816 (0.36)	0.612/0.430
Tubulolobular	P	1/115 (0.86)	27/8527 (0.32)	0.315/0.303
	B	0/21 (0.00)	3/501 (0.60)	1.000/0.723
	P+B	1/136 (0.73)	30/9028 (0.33)	0.373/0.424
DCIS with microinvasion	P	0/305 (0.00)	28/8337 (0.33)	0.625/0.312
	B	0/0 (0.00)	3/522 (0.57)	-
	P+B	0/305 (0.00)	31/8859 (0.35)	0.626/0.302
Molecular type of BC				
Oestrogen receptor-positive	P	16/5954 (0.27)	11/2591 (0.42)	0.293/0.240
	B	3/673 (0.44)	1/198 (0.50)	1.000/0.914
	P+B	19/6627 (0.29)	12/2789 (0.43)	0.324/0.269
Progesterone receptor-positive	P	14/5871 (0.24)	13/2372 (0.55)	**0.033/0.027**
HER2-positive	P	5/1265 (0.39)	20/5925 (0.34)	0.791/0.753
TNBC	P	6/1114 (0.54)	19/5798 (0.33)	0.277/0.285
Other high-risk features				
Bilateral BC	P	2/445 (0.45)	26/9314 (0.28)	0.368/0.513
	B	1/50 (1.96)	3/1405 (0.21)	0.133/**0.019**
	P+B	3/495 (0.60)	29/10719 (0.27)	0.167/0.173
BC diagnosed ≤40 y.o.	P	4/1282 (0.31)	30/11143 (0.27)	0.775/0.782
	B	1/353 (0.28)	3/1102 (0.27)	1.000/0.973
	P+B	5/1635 (0.30)	33/12245 (0.27)	0.799/0.792
≥1 BC/OC relatives	P	5/2186 (0.23)	28/9345 (0.30)	0.823/0.578
	B	1/428 (0.23)	3/1027 (0.29)	1.000/0.847
	P+B	6/2614 (0.23)	31/10372 (0.30)	0.683/0.553
Size (cm) of tumor				
<1	P	2/912 (0.22)	21/6974 (0.30)	1.000/0.667
1-1,9	P	9/3195 (0.28)	14/4691 (0.30)	1.000/0.893
2-4,9	P	10/3452 (0.29)	13/4434 (0.29)	1.000/0.977
≥5	P	2/327 (0.61)	21/7559 (0.28)	0.248/0.275
Other features				
Lymph node-positive	P	12/3539 (0.34)	12/4599 (0.26)	0.542/0.521
Vital status (deceased)	P	7/2059 (0.34)	26/10257 (0.25)	0.483/0.490

P, POLISH group; B, BELARUSIAN group; P+B, POLISH and BELARUSIAN group; HER2, human epidermal growth factor receptor 2; y.o., years old; M+, number of mutation carriers; M-, number of mutation noncarriers

Table S2. Characteristics of BC patients in terms of different clinical features and the status of p.R658C and p.R659R *BARD1* variants (carriers vs. noncarriers).

feature	**p.R658C**			**p.R659R**		
	**YES**	**NO**	**p-value**	**YES**	**NO**	**p-value**
	**M+/M- (%)**	**M+/M- (%)**	**Fisher/χ2**	**M+/M- (%)**	**M+/M- (%)**	**Fisher/χ2**
Histological type of BC						
Ductal, grade 3	10/1988 (0.50)	49/6623 (0.73)	0.351/0.265	6/1992 (0.30)	22/6650 (0.33)	1.000/0.839
Ductal, grade 1-2	28/4029 (0.69)	31/4582 (0.67)	1.000/0.918	15/4042 (0.37)	13/4600 (0.28)	0.570/0.472
Ductal, grade unknown	7/657 (1.05)	52/7954 (0.65)	0.216/0.223	1/663 (0.15)	27/7979 (0.34)	0.720/0.415
Medullary	2/285 (0.70)	57/8326 (0.68)	0.723/0.973	1/286 (0.35)	27/8356 (0.32)	0.611/0.938
Lobular	11/1232 (0.88)	48/7379 (0.65)	0.350/0.344	1/1242 (0.08)	27/7400 (0.36)	0.170/0.104
Tubulolobular	0/116 (0.00)	59/8495 (0.69)	1.000/0.370	2/114 (1.72)	26/8528 (0.30)	0.054/**0.007**
DCIS with microinvasion	1/304 (0.33)	58/8307 (0.69)	0.724/0.446	2/303 (0.66)	26/8339 (0.31)	0.259/0.297
Molecular type of BC						
Oestrogen receptor-positive	38/5932 (0.64)	21/2581 (0.81)	0.395/0.380	23/5947 (0.39)	8/2594 (0.30)	0.697/0.581
Progesterone receptor-positive	44/5841 (0.75)	12/2373 (0.50)	0.240/0.219	26/5859 (0.44)	4/2381 (0.17)	0.069/0.060
HER2-positive	8/1262 (0.63)	38/5907 (0.64)	1.000/0.970	4/1266 (0.31)	23/5922 (0.39)	1.000/0.703
TNBC	6/1114 (0.54)	38/5779 (0.65)	0.837/0.650	2/1118 (0.18)	24/5793 (0.41)	0.419/0.241
Other high-risk features						
Bilateral BC	4/443 (0.89)	61/9279 (0.65)	0.541/0.539	0/447 (0.00)	38/9302 (0.41)	0.419/0.177
BC diagnosed ≤40 y.o.	7/1279 (0.54)	73/11100 (0.65)	0.853/0.643	5/1281 (0.39)	44/11129 (0.39)	1.000/0.978
≥1 BC/OC relatives	14/2177 (0.64)	61/9312 (0.65)	1.000/0.951	3/2188 (0.14)	39/9334 (0.42)	**0.049**/0.051
Size (cm) of tumor						
<1	4/910 (0.44)	53/6942 (0.76)	0.403/0.282	5/909 (0.55)	20/6975 (0.29)	0.201/0.186
1-1,9	23/3181 (0.72)	34/4671 (0.72)	1.000/0.980	12/3192 (0.37)	13/4692 (0.28)	0.541/0.445
2-4,9	28/3434 (0.81)	29/4418 (0.65)	0.424/0.414	8/3454 (0.23)	17/4430 (0.38)	0.313/0.235
≥5	2/327 (0.61)	55/7525 (0.73)	1.000/0.805	0/329 (0.00)	25/7555 (0.33)	0.624/0.297
Other features						
Lymph node-positive	31/3520 (0.87)	26/4585 (0.56)	0.108/0.096	13/3538 (0.37)	12/4599 (0.26)	0.423/0.391
Vital status (deceased)	21/2045 (1.02)	59/10224 (0.57)	**0.034/0.022**	8/2058 (0.39)	41/10242 (0.40)	1.000/0.940

HER2, human epidermal growth factor receptor 2; y.o., years old; M+, number of mutation carriers; M-, number of mutation noncarriers

Table S3. The computational analyses of the *BARD1* variants selected for the analysis.

	AA change	p.Q564X	p.R658C	p.R659R
	nucleotide change	c.1690C>T	c.1972C>T	c.1977A>G
	dbSNP database id	rs587780021	rs3738888	rs147215925
	exon	8	10	10
	ACMG Classification[1]	Pathogenic [PVS1+PS3+PS4+PP3+PP5]	Unknown variant [BP1+BP6+PP1+PP3]	Unknown variant [PS3+PP3+BP6]
	ClinVar Classification	Pathogenic	Conflicting interpretations of pathogenicity Benign(6);Likely benign(3);Uncertain significance(2)	Conflicting interpretations of pathogenicity Benign(3);Likely benign(7);Uncertain significance(2)
predicted effect on splicing	MutPred Splice	splice affecting variant (SAV) - confident call of splicing variant (general score: 0.79)	splice neutral variant (SNV) (general score: 0.43)	splice affecting variant (SAV) - confident call of splicing variant (general score: 0.72)
	Human Splicing Finder<br>- influence on splicing[2]<br>- activation/inactivation of ESR elements[2]	creation of a new 5' cryptic site (122 nt upstream)<br>+SRp55 [1686_1691], -SF2/ASF (IgM-BRCA1) [1690-1696], -SF2/ASF [1690-1696], +ESS [1688-1695], +ESS [1687-1692], +IIE [1686-1691], -IIE [1687-1692]	disruption of potential branch point motif<br>+PESE [1972-1979], +EIE [1971-1976], +EIE [1972-1977], -9G8 [1967-1972], -ESS [1969-1976], +PESS [1968-1975], +ESR [1968-1973], +ESR [1970-1975]	disruption of potential branch point motif<br>+SRp40 [1971-1977], -ESE [1973-1978], -ESE [1974-1979], -ESE [1975-1980], -ESE [1976-1981], -ESE [1977-1982], -PESE [1975-1982], -EIE [1973-1978], -EIE [1974-1979], - 9G8 [1973-1978], +9G8 [1974-1979], -9G8 [1976-1981], +ESS [1974-1981], +ESS [1976-1983], +hnRNP A1 [1974-1979], -ESR [1973-1978]
	ESEfinder	+SRp55, -SF2/ASF (igM-BRCA1), -SF2/ASF	-SF2/ASF (igM-BRCA1), -SF2/ASF	-SRP55
	Rescue ESE	-	-	-ESE [1973-1978], -ESE [1974-1979], -ESE [1975-1980], -ESE [1976-1981], -ESE [1977-1982]
	Skippy	**analysis of changes in ESR:** association with HapMap SNPs (Log Odds Ratio Total = -3.894); **other variant-based features: analysis of a distance of the variant from a splice junction** - association with splice affecting variant (min distance as proportion of exon length 0.1955), **regulatory constraint (RC) score** - comparable to HapMap SNPs mean score (0.959); **exonic enviroment: splice junction strength** - strong 5' splice site (MaxEnt 5' splice site score: 10.07), strong 3' splice site (MaxEnt3' splice site score: 9), **exonic ESE density** - less than the mean HapMap SNPs exonic ESE density (score: 0.20), **exonic ESS density -** higher than the mean HapMap SNPs exonic ESS density (score: 0.21); **ectopic splice site variants analysis:** no potential to create ectopic splice sites (score: 0); **intronic enviroment - upstream intronic ESS density (100bp)** - higher than the mean HapMapSNPs intronic density (0.411), **downstream intronic ESS density (100 bp)** - higher than the mean HapMap SNPs intronic density (0.421)	**analysis of changes in ESR:** association with HapMap SNPs (Log Odds Ratio Total = -5.168); **other variant-based features: analysis of a distance of the variant from a splice junction** - score comperable to HapMapSNPs mean score, **regulatory constraint (RC) score** - association with splice neutral variant (0.533); **exonic enviroment: splice junction strength** -weak 5' splice site (MaxEnt 5'Splice Site score 3.5), strong 3' splice site (MaxEnt 3' splice site score: 6.81), **exonic ESE density** - higher than the mean HapMap SNPs exonic ESE density (score: 0.538), **exonic ESS density** - comparable to the mean HapMap SNPs ESS density (score: 0.086); **ectopic splice site variants analysis** - no potential to create ectopic splice sites (score: 0); **intronic enviroment: upstream intronic ESS density (100bp)** - comparable to the mean HapMap SNPs intronic density (0.337), **downstream intronic ESS density (100 bp)** - comparable to the mean HapMap SNPs intronic density (0.305)	**analysis of changes in ESR:** association with HapMap SNPs (Log Odds Ratio Total = -5.884); **other variant-based features: analysis of a distance of the variant from a splice junction** - score comperable to HapMap SNPs mean score; **regulatory constraint (RC) score** - comparable to HapMap SNPs mean score (1.246); **exonic enviroment: splice junction strength** - weak 5' splice site (MaxEnt 5'Splice Site score: 3.5), strong 3'splice site (MaxEnt 3'Splice Site score: 6.81), **exonic ESE density** - higher than the mean HapMapSNPs exonic ESE density (score: 0.538), **exonic ESS density** - comparable to the mean HapMapSNPs exonic ESS density (score: 0.086); **ectopic splice site variants analysis:** no potential to create ectopic splice sites (score: 0); **intronic enviroment: upstream intronic ESS density (100bp)** - comparable to the mean HapMapSNPs intronic density (0.337), **downstream intronic ESS density (100 bp)** - comparable to the mean HapMapSNPs intronic density (0.305)
	Spliceman	percentile rank (L1)= 77%	percentile rank (L1) = 67%	percentile rank (L1) = 60%
predicted effect on protein structure	MutPred	not applicable	probability of deleterious mutation: 0.154	not applicable
	LS-SNP	not applicable	highly confident prediction of disease-association, protein destabilization - desolubilizing effect	not applicable
	Pmut	not applicable	pathogenicity index: 0.897/1, confidence index:7/9, prediction: pathological	not applicable
	PolyPhen2	not applicable	probably damaging (score 0.995/1), R658 - weakly conserved between species	not applicable
	BLOSUM62	not applicable	score -3	not applicable
	PANTHER	not applicable	deleterious effect (subPSEC score -3.03044)	not applicable
	SIFT<br>- SIFT human protein<br>- SIFT / PROVEAN SNP	not applicable<br>not applicable	damaging<br>damaging/deleterious	not applicable<br>not applicable
	Mutation Assessor	not applicable	FI score: 1.335, functional impact: low	not applicable
	SNPper - amino acid variation	not applicable	major change in chemical nature: Arg positive polar <-> Cys uncharged polar; major change in alpha-helix propensity - Arg -0,68 kcal/mol <-> Cys -0.23 kcal/mol	not applicable
	PredictSNP 1.0	not applicable	deleterious (confidence 55%)	not applicable
evolutionary conservation	primate conservation by PhasCons (phastCons44wayPrimates)	moderate probability that the nucleotide belongs to a conserved element (score: 0,582307[3])	high probability that the nucleotide belongs to a conserved element (score: 0,936071[3])	high probability that the nucleotide belongs to a conserved element (score: 0,983268[3])
	primate basewise conservation by phyloP (phyloP44wayPrimate)	nucleotide site predicted to be conserved (score: 0,752795[3])	nucleotide site predicted to be not conserved (score: -0,614213[3])	nucleotide site predicted to be conserved (score: 0,801976[3])

Mutations are described based on BARD1 mRNA sequence (GenBank NM_000465.2), considering A of the first ATG- translation initiation codon as nucleotide 1, in consonance with Human Genome Variation Society (HGVS) (http://www.hgvs.org) nomenclature scheme; +, motif created by mutation, values in brackets indicate genomic position of a particular motif; -, motif disrupted by mutation, values in brackets indicate genomic position of a particular motif; [1], interpretation of variants pathogenicity based on the American the College of Medical Genetics and Genomics (ACMG) recommendations; [2], analysis of BARD1 ENST00000260947; [3], PhastCons and PhyloP scores extracted from UCSC Genome Browser tracks

Table S4. Primers and probes used for *BARD1* variants genotyping with the TaqMan assay.

The list of primers and probes	
**p.Q564X**	
5'-[HEX]CCATCCCTACGCT**G**CC[BHQ1]-3'	P1 WT
5'-[6FAM]CCATCCCTACGCT**A**CCCA[BHQ1]-3'	P2 MUT
5'-GCCCACTGCCTATAAGTACAAGAG-3'	F
5'-CACTGGTATCTCCTTTTATATTAACAGATG-3'	R
**p.R658C**	
5′-[HEX]TCCTGAAGGTCCA**C**GC[BHQ1]-3′	P1 WT
5′-[6FAM]TCCTGAAGGTCCA**T**GCA[BHQ1]-3′	P2 MUT
5'-AGTATGTGAACAGGAAGAAAAGTATGA-3'	F
5'-GTTGTATTAAAAGAAAAATACCAGCTG-3'	R
**p.R659R**	
5′-[HEX]TCTCTGTTGAGCCTGCT**T**CT[BHQ1]-3′	P1 WT
5′-[6FAM]CTCTGTTGAGCCTGCT**C**CT[BHQ1]-3′	P2 MUT
5'-AGTATGTGAACAGGAAGAAAAGTATGA-3'	F
5'-GTTGTATTAAAAGAAAAATACCAGCTG-3'	R

Table S5. Primers used for validation of *BARD1* variants.

**The list of primers**		
**p.Q564X**		
5′GGTTCTGGGTGTAGATTCA3′	F_outer	Tetra-primer ARMS-PCR assay
5′TACAAGATGCAAAGTATACAGCC3′	R_outer	
5′TTATATTAACAGATGAACACTGTGC3′	F_inner	
5′AGAGGTCCATCCCTACGATA3′	R_inner	
5′AGATGCCCTGGGTATAGAGA3′	F	Sanger sequencing
5′CCTCACCTGTACTGTCAAACT3′	R	
**p.R658C & p.R659R**		
5′GAGAGAGATATAGTGCTCACTTGA3′	F	Sanger sequencing
5′TGTTGAAAGGGCAGAAGTTC3′	R	
